# Exploring the relationship between transferrin and progression-free survival in breast cancer patients after neoadjuvant therapy

**DOI:** 10.3389/fonc.2026.1931384

**Published:** 2026-10-28

**Authors:** Jing He, Zhaoqi Zang, Jiaqin Zhou, Xinyu Wang, Yiying Shao, Guoqing Li, Shu Yang, Weifeng Shi, Ling Chen

**Affiliations:** 1 Affiliated Hospital of Jiangnan University, Jiangnan University, Wuxi, Jiangsu, China; 2 Wuxi Medical School, Jiangnan University, Wuxi, Jiangsu, China

**Keywords:** breast cancer, neoadjuvant chemotherapy, PFS (progression-free survival), survival analysis, transferrin

## Abstract

**Background:**

Since the first global cancer burden assessment report was released in the 1980s, breast cancer has consistently been the most frequently diagnosed malignancy in women. To this day, it remains the leading cause of cancer incidence and cancer-related mortality among women. Pathological evaluation of early breast cancer following neoadjuvant therapy is critical for providing prognostic information and guiding postoperative treatment decisions based on tumor response, including pathological complete response (pCR) and non-pCR status. Transferrin plays a central role in systemic iron metabolism, and dysregulated iron metabolism is a common hallmark of cancer. Therefore, exploring the relationship between transferrin and progression-free survival after neoadjuvant therapy for breast cancer can effectively predict the prognosis, thereby conferring positive clinical significance for subsequent treatment strategies. This study aimed to explore the association between transferrin and progression-free survival in breast cancer patients after neoadjuvant therapy.

**Method:**

**Research design:**

We conducted a retrospective study on breast cancer patients admitted to the Affiliated Hospital of Jiangnan University between 2021 and 2025, focusing on patients hospitalized in the Department of Breast Surgery who underwent surgical resection following neoadjuvant chemotherapy. After excluding patients who did not meet the inclusion criteria of this study, a total of 166 eligible cases were ultimately included. Multivariate Cox proportional hazards regression analysis was used to explore the association between preoperative transferrin and progression-free survival in breast cancer patients receiving neoadjuvant chemotherapy. Restricted cubic spline (RCS) curves were employed to evaluate the nonlinear relationship between transferrin levels and the hazard ratio of progression-free survival, and ROC curves were used to assess the ability of transferrin to distinguish between high and low prognosis of PFS.

**Result:**

This study included 166 patients with breast cancer. Multivariate Firth penalized Cox proportional hazards model analysis showed that transferrin (TRF) levels were independently associated with progression-free survival. The time-dependent AUC curve showed modest and fluctuating discriminative ability of TRF across the follow-up period, with values consistently ranging between 0.54 and 0.62 after the first year. Restricted cubic spline (RCS) analysis revealed an approximately linear monotonic increasing relationship between TRF and PFS (non-linearity test χ² = 1.46, P = 0.228). Kaplan-Meier survival curves demonstrated a clear distinction in progression-free survival between patients with high and low TRF levels.

**Conclusion:**

TRF was significantly associated with progression-free survival in patients with neoadjuvant breast cancer. However, due to the limitations of this study, we are only doing preliminary exploration for information, and do not make any causal inference. A larger cohort is needed to explore in the future.

## Introduction

Transferrin (Tf) is an 80 kDa monomeric glycoprotein composed of two homologous lobes (N-lobe and C-lobe) connected by a short peptide chain (1). Each lobe consists of two subdomains (N1/N2 and C1/C2), which are linked by antiparallel β-sheets to form a flexible hinge. The cleft between the two lobes enables iron ion binding (2). Transferrin binds two ferric ions (Fe³^+^) with high affinity and requires carbonate as a ligand to stabilize the iron->binding sites (3).

Existing studies have demonstrated that transferrin plays a central role in systemic iron metabolism (4). Its main functions include solubilizing insoluble free Fe³^+^ under neutral pH conditions, transporting iron among sites of absorption, utilization and storage, inhibiting the production of reactive oxygen species (ROS), chelating toxic free iron to act as a protective scavenger, and participating in innate immunity by sequestering iron to restrict bacterial survival (5). Transferrin receptor (TRF)-mediated endocytosis represents the primary route for iron acquisition in most cells. Under acidic conditions (pH 5.6), the binding affinity between iron and transferrin declines, thereby facilitating iron release from the binding sites and subsequent intracellular entry via endocytosis (6). Four types of transferrin receptors have been identified to date: transferrin receptor 1 (TfR1), TfR2, TfR3 (glyceraldehyde-3-phosphate dehydrogenase), and TfR4 (cubilin). They function in distinct cells, organs and developmental stages to ensure adequate iron supply for cells and tissues (6). Among them, TfR1 is a key protein for cellular iron uptake. Encoded by the TRFC gene, it predominantly exists as a homodimer (7).

TfR1 is widely overexpressed in a variety of cancers with complex and interconnected regulatory mechanisms. Tumor cells upregulate TfR1 expression primarily to meet the excessive iron demand for rapid proliferation (8). Accumulating evidence indicates that TfR1 functions not only as an iron uptake receptor but also as a signaling molecule. For instance, phosphorylation of tyrosine 20 on TfR1 by Src kinase enhances anti->apoptotic effects and promotes the survival of breast cancer cells (9). TfR1 also acts as a mitochondrial regulator to facilitate cancer cell growth by activating the JNK signaling pathway (8). TfR1 plays a vital role in various types of cancers. For example, in pancreatic cancer, TfR1 drives tumor growth by supporting ROS generation and mitochondrial respiration. Its upregulated expression induces ROS production in pancreatic ductal adenocarcinoma cells through enhancing oxidative phosphorylation, and mitochondrial-derived ROS is essential for pancreatic cancer progression (10).

Accordingly, dysregulated iron metabolism is a common hallmark of cancer. Tumor cells effectively enhance iron uptake and retention through multiple adaptive strategies, including TfR1 overexpression, upregulation of STEAP proteins, and downregulation of ferroportin, thereby constructing an iron->rich microenvironment to sustain their aggressive proliferation and survival (11). Under hypoxic conditions, activation of the HIF signaling pathway augments iron uptake and utilization to meet the iron demand of rapidly proliferating tumor cells. This process not only facilitates tumor growth but also enhances tumor invasiveness and therapeutic resistance (12). Based on the established role of TfR1 as the primary portal for cellular iron uptake, serum transferrin (TRF) is its core ligand and iron donor for mediating endocytosis. Therefore, circulating TRF levels directly determine the iron flux that tumor cells obtain via the TfR1 axis. When serum TRF is elevated, it can saturate and activate the TfR1-mediated endocytic pathway, continuously delivering iron ions to tumor cells, promoting intracellular iron accumulation, thereby driving malignant proliferation, inhibiting ferroptosis, and activating pro-survival signaling cascades such as PI3K-AKT-mTOR; conversely, decreased TRF levels reflect systemic iron restriction and protein-energy malnutrition, which may both impair the supply of iron metabolism substrates to tumor cells and compromise the host’s tolerance and repair capacity for cytotoxic therapy (13). In summary, serum transferrin may bidirectionally influence tumor progression and treatment outcomes by regulating the TfR1-dependent iron uptake pathway, thereby affecting the patient’s PFS, and thus serving as a systemic surrogate marker reflecting TfR1-driven tumor metabolic activity.

Epidemiological data indicate that breast cancer is the most common malignant tumor in women worldwide and the second leading cause of cancer-related mortality (14). Despite the advances in early detection techniques and improved therapeutic regimens that have gradually reduced breast cancer mortality, neoadjuvant chemotherapy (NAC) remains a core therapeutic strategy for the management of locally advanced breast cancer (15).The expression of transferrin receptor 1 (TfR1) possesses crucial clinical significance in breast cancer (16). Comprehensive bioinformatic analyses have demonstrated that TfR1 is significantly upregulated in breast cancer tissues, and its high expression is closely correlated with multiple clinicopathological parameters, including high tumor grade, lymph node metastasis, hormone receptor negativity, and HER2 positivity (9). As early as 2010, a study reported that transferrin receptor (CD71) serves as a biomarker for poor prognosis in breast cancer and can be used to predict the therapeutic response to tamoxifen (7). However, the association between transferrin and progression-free survival following neoadjuvant therapy for breast cancer remains poorly investigated. Therefore, the present study is the first to reveal the relationship between transferrin and progression-free survival in breast cancer patients receiving neoadjuvant chemotherapy.

## Materials and methods

This study was designed as a retrospective analysis, enrolling breast cancer patients admitted to the Affiliated Hospital of Jiangnan University between 2021 and 2025. The research subjects included patients hospitalized in the Department of Breast Surgery who received neoadjuvant chemotherapy followed by radical mastectomy. This study aimed to explore the correlation between transferrin (TRF) level and progression-free survival in breast cancer patients, as well as its prognostic value across different molecular subtypes. Progression-free survival (PFS) refers to the time interval from the patient’s randomization or initiation of treatment to the occurrence of objective radiological tumor progression or death from any cause, whichever occurs first, and is a common clinical endpoint for evaluating the disease control effect of anti-tumor therapy.

All patients underwent pathological evaluation after neoadjuvant chemotherapy, and complete medical records were systematically preserved in the hospital. This study was approved by the Ethics Committee of the Affiliated Hospital of Jiangnan University. All data were fully anonymized; thus, written informed consent was waived. All research procedures were performed in accordance with relevant guidelines and regulations, including the Declaration of Helsinki. Clinical information (Including blood test results) was collected prior to initial surgery (after the completion of neoadjuvant therapy), and peripheral blood samples were obtained from patients after an 8-hour overnight fasting period (6:00–8:00 a.m.). However, patients were excluded from the study if they met any of the following criteria: (1) patients younger than 18 years or older than 80 years, (2) patients with a history of malignancy, (3) patients with non-primary breast cancer, (4) patients with unclear or rare/special pathology, (5) patients who did not complete neoadjuvant therapy per protocol, (6) patients with unclear treatment regimen, (7) patients who did not undergo post-treatment surgery, (8) patients with severely missing baseline data, and (9) populations who did not meet or complete a complete neoadjuvant regimen. The specific numbers of included and excluded patients are shown in **Figure 1**. All 166 patients had complete data for the variables included in the final model. Therefore, no complete-case exclusion was performed. Censoring occurred solely due to administrative censoring at the end of the study period, with no loss to follow-up. This study was designed and reported in accordance with the Strengthening the Reporting of Observational Studies in Epidemiology (STROBE) statement.

**Figure 1:**
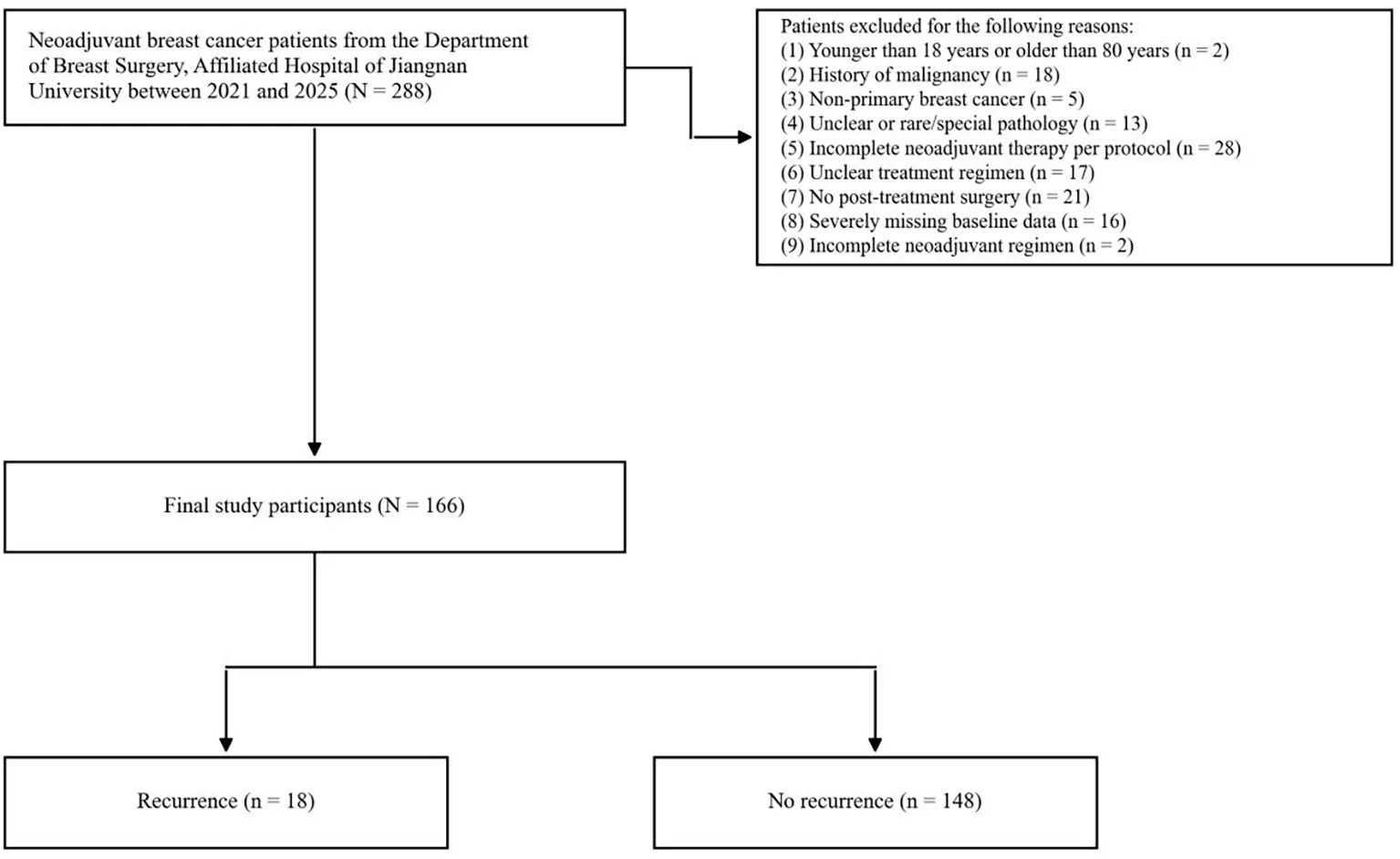
Patient enrollment flow chart.

We retrospectively reviewed medical records of enrolled patients and extracted demographic characteristics, pathological data, and laboratory examination results. The molecular subtypes and neoadjuvant treatment regimens of all breast cancer patients were determined in accordance with the Chinese Society of Clinical Oncology (CSCO) Guidelines for the Diagnosis and Treatment of Breast Cancer (17). According to the immunohistochemical results of diagnostic biopsy, patients with estrogen receptor (ER) or progesterone receptor (PR) expression levels ≥ 1% were defined as hormone receptor-positive. HER2 status should be determined by combining IHC and FISH testing. HER2 positivity is defined as IHC 3+, or IHC 2+ with positive FISH/ISH; HER2 negativity is defined as IHC 0 or 1+, or IHC 2+ with negative FISH/ISH. Breast cancer is divided into four subtypes defined as follows: Luminal A-like (ER positive, PR high expression, HER2 negative, Ki67 ≤14%), Luminal B-like (ER positive, HER2 negative but Ki67 >14%, or ER positive and HER2 positive), HER2-enriched (HER2 positive, ER negative), and triple-negative breast cancer (TNBC) (ER, PR, and HER2 all negative) (18).

Postoperative pathological response to neoadjuvant therapy was assessed by specialized pathologists using the Miller–Payne (MP) grading system. Pathological complete response (pCR) was defined as Miller-Payne grade 5 (no invasive carcinoma of the breast, regardless of residual ductal carcinoma *in situ*) and axillary lymph node negative (ypN 0) (19). In this study, the preoperative neoadjuvant treatment response of all patients was assessed using the Miller-Payne (MP) grading system determined by postoperative professional pathologists.

The hospital clinical laboratory uses the Mindray semi-automatic biochemistry analyzer BA-88A to detect total transferrin (TRF) levels, employing the immunoturbidimetric method, with a standard reference range of 2.2 to 4.0 g/L. We retrospectively analyzed the levels of complete blood count and biochemical markers, including PA, TRF, ALT, AST, albumin, LDH, TG, CHOL, HDL-C, LDL-C, GLU, CEA, CA15-3, WBC, neutrophils, maximum tumor diameter, lymphocytes, HB and PLT. Among these, lymph node status and maximum tumor diameter are indicators before neoadjuvant therapy, while the remaining indicators are after the completion of neoadjuvant therapy.

Hormone receptor-positive/human epidermal growth factor receptor 2-negative (HR+/HER2-) patients received epirubicin plus cyclophosphamide (EC, q3w × 4 cycles) followed by docetaxel (T, q3w × 4 cycles). Patients with HER2-positive breast cancer (including the HER2-enriched subtype and the HER2-positive subset of Luminal B-like) received docetaxel (75 mg/m², q3w × 6 cycles) in combination with carboplatin (AUC 6, q3w) and trastuzumab plus pertuzumab dual-target therapy (TCHP), or alternatively, epirubicin plus cyclophosphamide followed by trastuzumab plus pertuzumab (EC-THP). Triple-negative breast cancer patients received dose-intensive epirubicin plus cyclophosphamide (q2w × 4 cycles) followed by weekly paclitaxel (qw × 12 weeks) or dose-intensive paclitaxel (q2w × 4 cycles).

### Data collection

Data on participants’ transferrin (TRF, g/L), prealbumin (PA, mg/L), age, alanine aminotransferase (ALT, U/L), aspartate aminotransferase (AST, U/L), albumin(g/L), lactate dehydrogenase (LDH, U/L), triglycerides (TG, mmol/L), cholesterol (CHOL, mmol/L), high-density lipoprotein cholesterol (HDL-C, mmol/L), low-density lipoprotein cholesterol (LDL-C, mmol/L), glucose (GLU, mmol/L), carcinoembryonic antigen (CEA, ng/mL), carbohydrate antigen 153 (CA15-3, U/mL), white blood cell count(*10^9^/L), neutrophil count(*10^9^/L), lymphocyte count(*10^9^/L), hemoglobin level(Hb,g/L), and platelet count(*10^9^/L) were collected from the hospital information system (HIS).

### Statistical analysis

First, the Shapiro-Wilk test was used to assess the normality of all continuous variables. Continuous variables with normal distribution were described as mean (standard deviation), and comparisons between groups were performed using the independent samples t-test; continuous variables with non-normal distribution were described as median (interquartile range, M [Q_1_, Q_3_]), and comparisons between groups were performed using the Mann-Whitney U test. Categorical variables were described as frequency (percentage), and comparisons between groups were performed using Fisher’s exact test. For the comparison of baseline characteristics, a P value < 0.05 was considered statistically significant.

Multivariable Firth-penalized Cox proportional hazards model was performed to explore the association between transferrin level and progression->free survival in breast cancer patients receiving neoadjuvant therapy. To account for censoring and evaluate the dynamic predictive performance of baseline serum transferrin (TRF) over the follow-up period, we performed time-dependent ROC curve analysis using the cumulative/dynamic approach proposed by Heagerty and Zheng. For a given time point t, patients who experienced disease progression or death at or before t were defined as cases, whereas those who remained event-free with follow-up longer than t were defined as controls. Patients censored before t were excluded from the ROC calculation at that specific time point because their event status could not be determined. Sensitivity and specificity were calculated accordingly, and the area under the ROC curve (AUC) was reported for each evaluated time point. This method provides a more accurate assessment of predictive accuracy than standard binary ROC analysis in the presence of censored survival data. A firth penalized restricted cubic spline (RCS) model was used to assess the nonlinear relationship between TRF and PFS. Survival curves were plotted via the Kaplan–Meier method, and the Log->rank test was used for intergroup comparison. The Cox proportional hazards model was applied to calculate hazard ratios (HRs) and corresponding 95% confidence intervals. A two-tailed P value < 0.05 was considered statistically significant, and P < 0.10 was defined as marginally significant. All statistical analyses were conducted using R software (version 4.3.1).

## Result

### Inclusion of patient baseline information

A total of 166 patients were included in this study (**Table 1**), 97 of whom had MP Grade of non-pCR, accounting for 58.43%, and 69 of whom had pCR, accounting for 41.57%. There were significant differences in ER, LDH, Hb and molecular typing between the two groups (P<0.05), but there were no significant differences in Age, PA, TRF, Ki67, maximum tumor diameter, Age, ALT, AST, Albumin, TG, CHOL, HDL-C, LDL-C, GLU, CEA, CA15-3, WBC, Neutrophill, Lymphocyte, Platelet and lymph node metastasis (P>0.05). During the follow-up period, a total of 18 patients experienced cancer recurrence, with follow-up durations ranging from 100 to 1940 days and a median follow-up time of 933 days.

**Table 1:** Baseline characteristics and intergroup difference analysis.

Variables	Total (n = 166)	Non-pCR (n = 97)	pCR (n = 69)	*P*
Age, M (Q_1_, Q_3_)	56.00 (50.00, 61.00)	56.00 (49.00, 61.00)	56.00 (50.00, 59.00)	0.693
PA, Mean ± SD	234.34 ± 45.84	235.33 ± 41.93	232.94 ± 51.13	0.741
TRF, Mean ± SD	2.19 ± 0.43	2.20 ± 0.44	2.18 ± 0.42	0.771
Ki67, M (Q_1_, Q_3_)	50.00 (40.00, 70.00)	50.00 (40.00, 70.00)	55.00 (40.00, 70.00)	0.615
ER, M (Q_1_, Q_3_)	30.00 (0.00, 90.00)	70.00 (0.00, 90.00)	0.00 (0.00, 70.00)	0.001
Maximum tumor diameter, M (Q_1_, Q_3_)	3.15 (2.40, 4.30)	3.20 (2.40, 4.30)	3.10 (2.30, 4.30)	0.557
ALT, M (Q_1_, Q_3_)	19.00 (14.00, 28.00)	19.00 (15.00, 29.00)	18.00 (13.00, 25.00)	0.159
AST, M (Q_1_, Q_3_)	22.00 (18.00, 29.00)	22.00 (19.00, 30.00)	21.00 (18.00, 28.00)	0.216
Albumin, M (Q_1_, Q_3_)	38.75 (36.82, 41.45)	38.80 (37.00, 41.20)	38.70 (36.70, 41.50)	0.805
LDH, M (Q_1_, Q_3_)	192.50 (168.00, 222.00)	201.00 (170.00, 231.00)	183.00 (157.00, 210.00)	0.036
TG, M (Q_1_, Q_3_)	1.48 (1.11, 2.04)	1.60 (1.11, 2.21)	1.38 (1.11, 1.81)	0.124
CHOL, M (Q_1_, Q_3_)	4.79 (4.26, 5.42)	4.83 (4.37, 5.38)	4.66 (4.12, 5.62)	0.417
HDL-C, M (Q_1_, Q_3_)	1.13 (0.99, 1.35)	1.17 (1.02, 1.34)	1.10 (0.97, 1.37)	0.319
LDL-C, M (Q_1_, Q_3_)	3.08 (2.70, 3.50)	3.15 (2.72, 3.56)	3.03 (2.62, 3.48)	0.467
GLU, M (Q_1_, Q_3_)	5.13 (4.69, 5.73)	5.06 (4.68, 5.64)	5.21 (4.77, 5.81)	0.421
CEA, M (Q_1_, Q_3_)	1.09 (0.96, 1.69)	1.21 (1.00, 1.75)	1.01 (0.88, 1.50)	0.088
CA15-3, M (Q_1_, Q_3_)	8.25 (7.00, 12.08)	8.30 (7.00, 14.00)	8.09 (7.00, 10.50)	0.266
WBC, M (Q_1_, Q_3_)	4.50 (3.80, 5.57)	4.50 (3.60, 5.70)	4.50 (3.90, 5.40)	0.825
Neutrophil, M (Q_1_, Q_3_)	2.80 (2.00, 3.70)	2.80 (2.10, 3.70)	2.80 (2.00, 3.80)	0.672
Lymphocyte, M (Q_1_, Q_3_)	1.20 (1.00, 1.48)	1.10 (0.90, 1.40)	1.30 (1.00, 1.50)	0.053
Hb, M (Q_1_, Q_3_)	109.00 (102.00, 119.00)	114.00 (104.00, 121.00)	106.00 (98.00, 114.00)	0.006
Platelet, M (Q_1_, Q_3_)	213.00 (167.25, 257.75)	207.00 (162.00, 245.00)	216.00 (185.00, 259.00)	0.519
Molecular typing, n(%)				0.002
TNBC	34 (20.48)	21 (21.65)	13 (18.84)	
HER2-Enriched	34 (20.48)	11 (11.34)	23 (33.33)	
Luminal A-like	10 (6.02)	9 (9.28)	1 (1.45)	
Luminal B-like	88 (53.01)	56 (57.73)	32 (46.38)	
Lymphatic metastasis, n(%)				0.642
No	14 (8.43)	9 (9.28)	5 (7.25)	
Yes	152 (91.57)	88 (90.72)	64 (92.75)	

### The relationshap between TRF and progression-free survival in post-neoadjuvant breast cancer

In this study, among the enrolled patients, only 18 experienced recurrence during follow-up. To avoid the finite-sample bias of Cox regression under small samples, a multivariable Firth-penalized Cox proportional hazards model was used to estimate the independent association between TRF and PFS. The Firth method introduces Jeffreys prior into the Cox partial likelihood function for penalization, effectively reducing regression coefficient bias in scenarios with small samples and low event rates. The model included four covariates strongly associated with the prognosis of breast neoplasms: age, maximum tumor diameter, pCR status, and Molecular subtyping (Using HER2-enriched type as reference) (20–22), to avoid overfitting caused by excessive variables. The proportional hazards (PH) assumption was tested using the Schoenfeld residual method.

Schoenfeld residual test showed that none of the five covariates violated the proportional hazards assumption (TRF: ρ = 0.273, P = 0.272; all other variables P > 0.30), indicating that the Cox model specification was appropriate (**Table 2**). In the multivariable Firth penalized Cox proportional hazards model (**Table 3**), after adjusting for age, maximum tumor diameter, pathological complete response status, and molecular typing, baseline TRF levels were significantly associated with progression-free survival (β = 1.226, SE = 0.598, HR = 3.41, 95% CI: 1.06–10.99, P = 0.040). The multicollinearity check revealed no apparent multicollinearity among the variables (**Table 4**). The results suggest that TRF may be a Potential prognostic factor for PFS. However, due to the limited sample size in this study, the current findings can only be considered exploratory and do not prove any causal relationship.

**Table 2:** Schoenfeld residuals test.

Variables	Rho	P
Age	-0.121	0.633
Tumor size	0.154	0.542
pCR	-0.253	0.311
Luminal	-0.013	0.958
TNBC	0.157	0.537
TRF	0.273	0.272

**Table 3:** Multivariable Firth-penalized Cox proportional hazards model.

Variables	β (Firth)	SE	HR	95%CI	P
Age	0.034	0.027	1.03	0.98-1.09	0.211
Tumor size	0.095	0.118	1.10	0.87-1.38	0.422
pCR	-0.312	0.518	0.73	0.27-2.02	0.547
Molecular subtyping					
HER2(+)	Reference	Reference	Reference	Reference	Reference
Luminal	-0.171	0.691	0.84	0.22-3.26	0.804
TNBC	0.879	0.703	2.41	0.61-9.54	0.211
TRF	1.226	0.598	3.41	1.06-10.99	0.040

**Table 4:** Variance inflation factor (VIF) analysis.

Variable	VIF
Age	1.253
Tumor size	1.039
pCR	1.088
Luminal	1.730
TNBC	1.658
TRF	1.258

Time-dependent ROC analysis (**Figure 2**) was performed to evaluate the dynamic predictive accuracy of baseline TRF for progression-free survival (PFS) while appropriately handling censored observations. Using the cumulative/dynamic approach, the AUC values at 1, 2, and 3 years were 0.609, 0.549, and 0.616, respectively. The time-dependent AUC curve showed modest and fluctuating discriminative ability of TRF across the follow-up period, with values consistently ranging between 0.54 and 0.62 after the first year. The time-dependent predictive performance of TRF is summarized in **Figure 3**.

**Figure 2:**
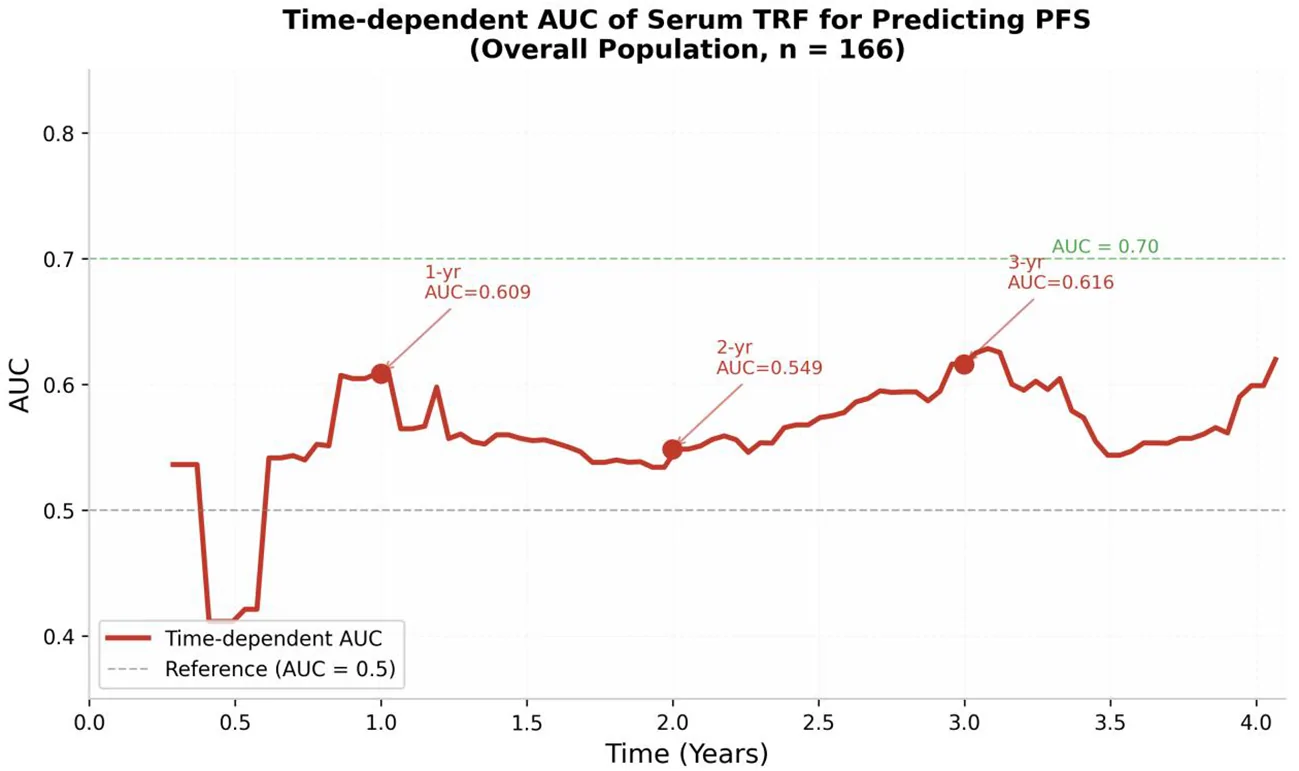
Receiver operating characteristic (ROC) curve analysis.

**Figure 3:**
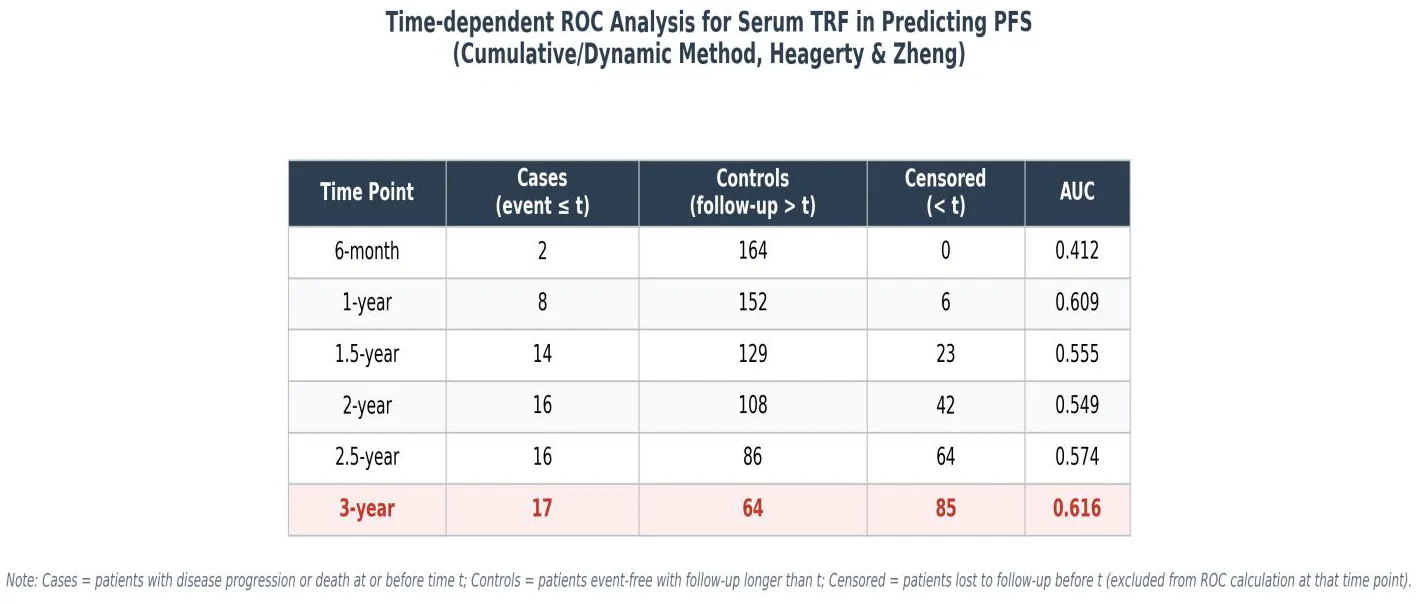
Time-dependent ROC analysis of baseline serum transferrin for predicting progression-free survival at different time points.

After adjusting for age, maximum tumor diameter, pCR and molecular subtyping, a Firth-penalized RCS was used to fit the dose-response association between TRF and PFS. The restricted cubic spline (RCS) analysis (**Figure 4**) showed an approximately linear monotonic increasing relationship between TRF and PFS (nonlinearity test χ² = 1.46, P = 0.228).

**Figure 4:**
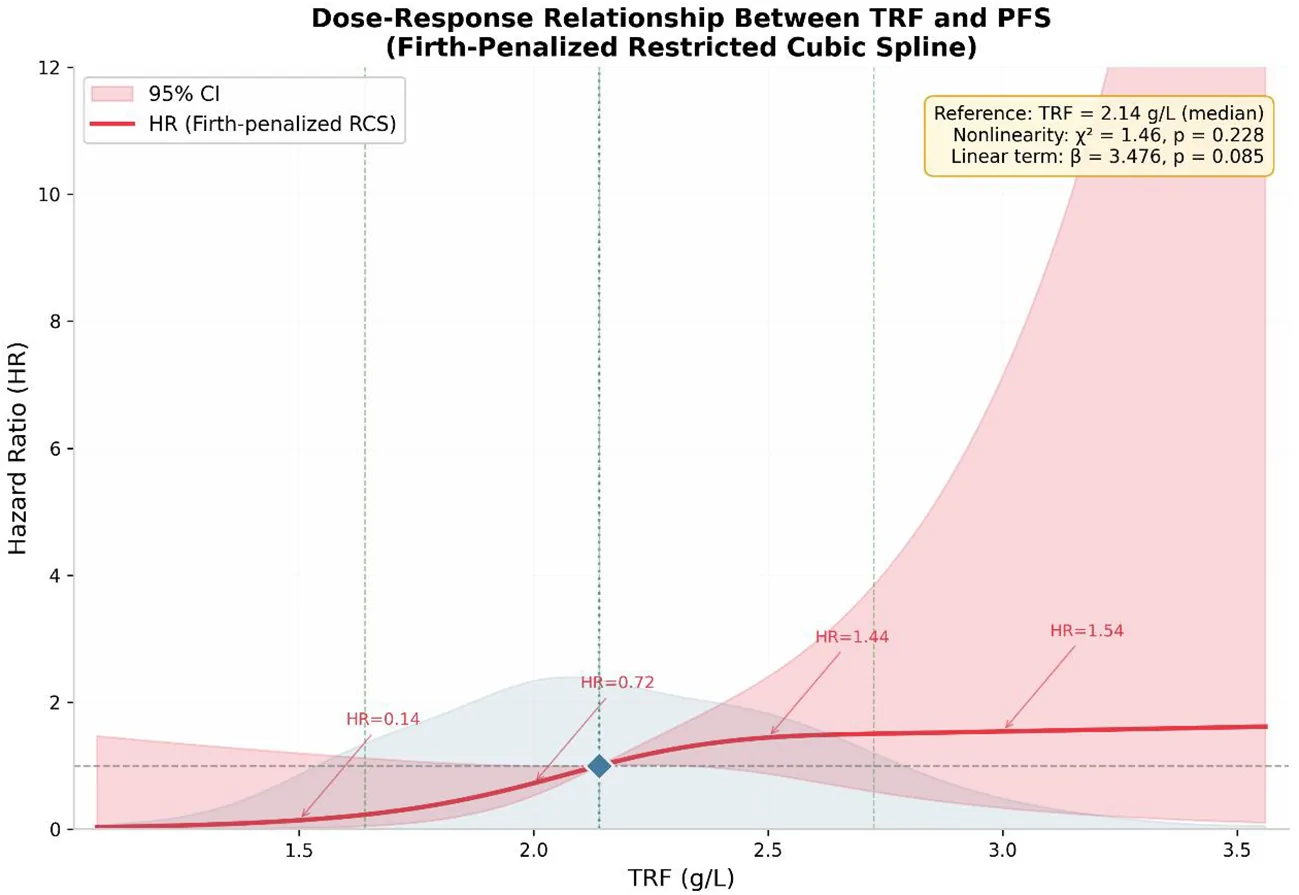
Restricted cubic spline (RCS) analysis.

### KM survival curve analysis of TRF at different levels and breast cancer PFS

Kaplan-Meier analysis (**Figure 5**) stratified by TRF median (2.14 g/L) showed that patients with high TRF had consistently lower progression-free survival probabilities than those with low TRF, with the curves beginning to diverge after approximately 500 days and the gap widening toward the end of follow-up (Log-rank χ² = 6.01, P = 0.014).

**Figure 5:**
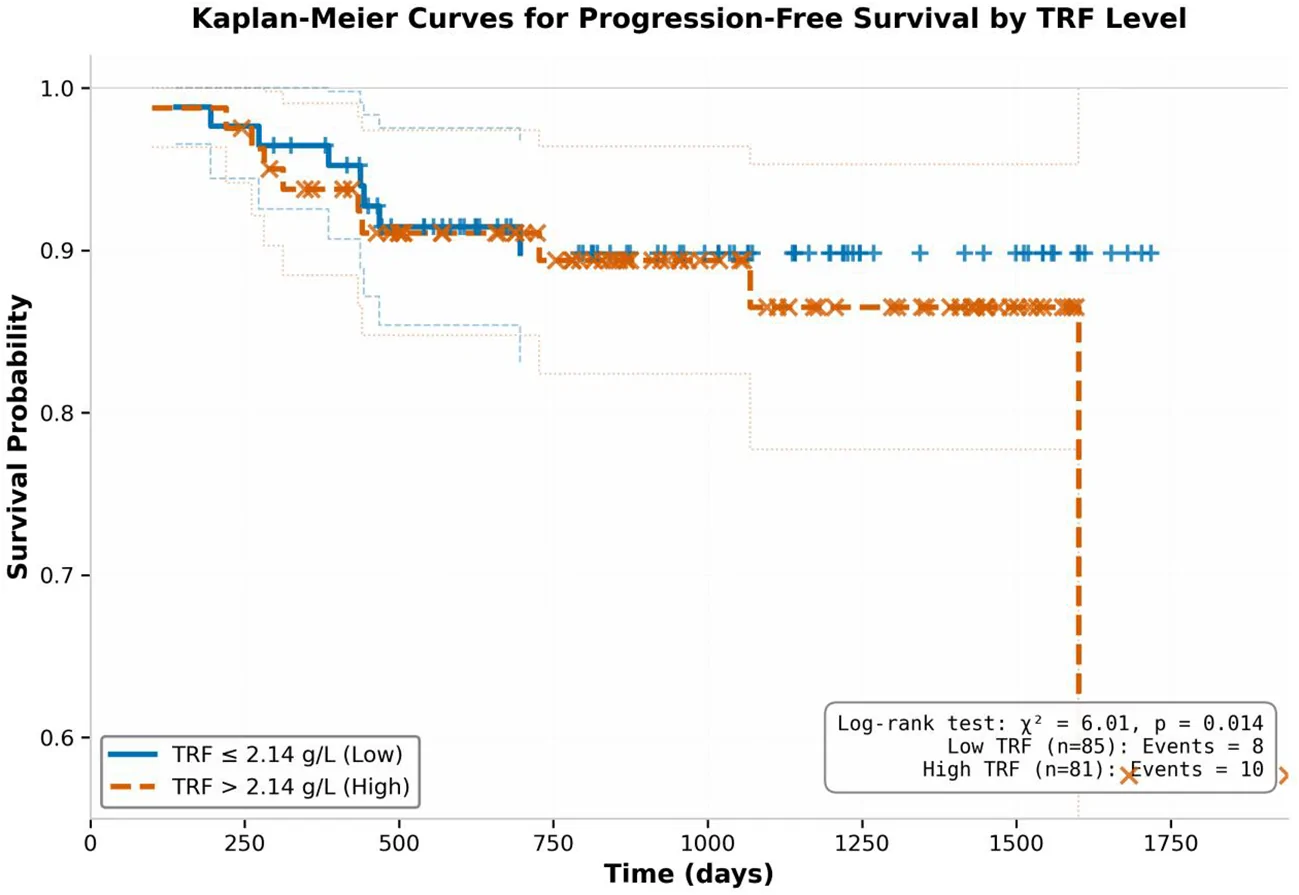
Kaplan–Meier survival curves for PFS by TRF level.

## Discussion

This study systematically evaluated for the first time the association between baseline serum transferrin (TRF) and progression-free survival (PFS) in 166 breast cancer patients receiving neoadjuvant chemotherapy. Multivariable Firth penalized Cox regression analysis showed that baseline TRF levels were potentially associated with PFS after adjusting for age, tumor size, pCR status, and molecular subtypes (HR = 3.41, 95% CI: 1.06–10.99, P = 0.040), suggesting that TRF may be a potential prognostic marker for breast cancer patients undergoing neoadjuvant therapy. Time-dependent ROC analysis further indicated that TRF’s dynamic predictive performance for PFS was relatively low, with AUC values of 0.609, 0.549, and 0.616 at 1, 2, and 3 years, respectively. The RCS curve indicates an overall increasing trend between TRF and PFS. Kaplan-Meier analysis confirmed that PFS in the high TRF group was significantly worse than that in the low TRF group (Log-rank P = 0.014), with the two curves beginning to separate after approximately 500 days of follow-up and diverging over time.

Based on our findings and existing research, we speculate that Transferrin may influence the development of breast tumors through the following mechanisms. Firstly, Iron metabolism plays a critical role in breast cancer progression. Breast cancer cells enhance iron uptake via overexpression of transferrin receptor 1 (TfR1), leading to intracellular iron accumulation, which facilitates rapid cell proliferation, metastasis, and therapeutic resistance (23). Elevated ferritin levels further protect tumor cells from oxidative stress damage, while reduced expression of ferroportin restricts iron efflux, thereby creating an iron-rich microenvironment conducive to tumorigenesis and tumor progression (23). These alterations in iron homeostasis are closely associated with aggressive cancer phenotypes and adverse clinical outcomes. In estrogen receptor-positive breast cancer, estrogen modulates iron metabolism by upregulating TfR1 expression, which enhances cellular iron uptake and storage. This evidence indicates a tight correlation between iron metabolism and hormone-driven tumor progression (23).

Moreover, in the high TRF range, elevated circulating TRF indicates abundant systemic iron reserves. Enhanced iron uptake mediated by transferrin receptor 1 (TfR1) supplies essential iron for tumor cell proliferation and simultaneously inhibits ferroptosis, a key cell death modality that contributes to chemotherapy sensitivity (24). Especially in the TNBC subtype, tumor cells frequently overexpress TfR1 and exhibit strong dependence on iron metabolism. High TRF levels continuously supply iron ions, activate the PI3K->AKT->mTOR signaling pathway to facilitate tumor progression, and ultimately induce resistance to neoadjuvant chemotherapy (25). In addition, iron chelators such as deferoxamine can enhance chemosensitivity in TNBC by inducing ferroptosis, which indirectly validates the pivotal role of the TRF->iron axis in this specific subtype (26). Moreover, the change in TRF before and after treatment may carry additional predictive value. Chemotherapy itself can alter iron homeostasis, erythropoiesis, and hepatic protein synthesis, and ΔTRF could therefore theoretically reflect both tumor regression and host tolerance.

However, the above are all speculations based on the mechanisms studied so far. Currently, there are no large-scale studies directly confirming that TRF levels directly intervene in the development of breast cancer after neoadjuvant therapy, which also points a direction for future research.

In summary, TRF may be a valuable hematological marker for the prognosis of breast cancer patients undergoing neoadjuvant therapy, but its clinical application value still needs to be further validated in large-scale, multicenter prospective cohorts. Future research should focus on the combined assessment of TRF and TfR1 expression levels, as well as the potential role of iron metabolism intervention strategies in improving the prognosis of neoadjuvant chemotherapy. Any results should not be overinterpreted as causal evidence.

## Conclusion

This study revealed a significant association between baseline serum transferrin (TRF) and progression-free survival in breast cancer patients undergoing neoadjuvant chemotherapy. Multivariate analysis indicated that TRF is a potential prognostic factor for progression-free survival (PFS). Restricted cubic spline (RCS) analysis showed an approximately linear increasing relationship between TRF and recurrence risk.

## Study limitation

First, as a single-center retrospective study, the sample size was limited (n = 166), and the number of events was small (18 cases of recurrence), resulting in insufficient statistical power and limited estimation precision (e.g., the HR 95% CI for TRF had a wide range). Second, the study simultaneously conducted multiple statistical inferences, including Cox regression analysis, ROC, RCS, KM analysis, and subgroup comparisons, but did not perform multiple comparison corrections, posing a risk of inflated Type I error. Third, the time-dependent ROC indicated that the discriminative ability of TRF was low, and the nonlinearity test was not significant (P = 0.147). Moreover, the change in serum TRF before and after treatment (ΔTRF) may carry additional predictive value; however, in this retrospective cohort, pre-neoadjuvant TRF was not routinely measured and was missing for a substantial proportion of patients, precluding calculation of ΔTRF. Finally, the retrospective design cannot establish a causal relationship, and the association between TRF and PFS may be influenced by unmeasured confounding factors (e.g., inflammatory status, nutritional scores, etc.). Our current inferences are only preliminary explorations, and any conclusions from this study should not be overinterpreted.

## Data Availability

The raw data supporting the conclusions of this article will be made available by the authors, without undue reservation.

## References

[1] Cao J . Functional divergence of the N-lobe and C-lobe of *Transferrin* gene in *Pungitius sinensis* (Amur Stickleback). Anim (Basel). (2022) 12(24):3458. doi: 10.3390/ani12243458 36552378 PMC9774405

[2] Chesnokov Y Mozhaev A Kamyshinsky R Gordienko A Dadinova L . Structural insights into iron ions accumulation in Dps nanocage. Int J Mol Sci. (2022) 23(10):5313. doi: 10.3390/ijms23105313 35628121 PMC9140674

[3] Borko V Friganović T Weitner T . Glycoproteomics meets thermodynamics: A calorimetric study of the effect of sialylation and synergistic anion on the binding of iron to human serum transferrin. J Inorg Biochem. (2023) 244:112207. doi: 10.1016/j.jinorgbio.2023.112207 37054508

[4] Fu X Feng Y Wu Z Qin Y Qin H Yao K . Transferrin receptor 1: An emerging therapeutic target in cancer beyond iron metabolism. Cancer Cell Int. (2026) 26(1):290. doi: 10.1186/s12935-026-04380-6 42286679 PMC13488156

[5] Cain TJ Smith AT . Ferric iron reductases and their contribution to unicellular ferrous iron uptake. J Inorg Biochem. (2021) 218:111407. doi: 10.1016/j.jinorgbio.2021.111407 33684686 PMC8035299

[6] Guo Q Qian C Wang X Qian ZM . Transferrin receptors. Exp Mol Med. (2025) 57:724–32. doi: 10.1038/s12276-025-01436-x 40263550 PMC12045970

[7] Candelaria PV Leoh LS Penichet ML Daniels-Wells TR . Antibodies targeting the transferrin receptor 1 (TfR1) as direct anti-cancer agents. Front Immunol. (2021) 12:607692. doi: 10.3389/fimmu.2021.607692 33815364 PMC8010148

[8] Wang Z Liu J Chen H Qiu X Xie L Kaniskan HÜ . Telomere targeting chimera enables targeted destruction of telomeric repeat-binding factor proteins. J Am Chem Soc. (2023) 145:10872–9. doi: 10.1021/jacs.3c02783 37141574 PMC10976431

[9] Habib I Khan S Mohammad T Hussain A Alajmi MF Rehman T . Impact of non-synonymous mutations on the structure and function of telomeric repeat binding factor 1. J Biomol Struct Dyn. (2022) 40:9053–66. doi: 10.1080/07391102.2021.1922313 33982644

[10] Yang C Dong Q Bao H Ge Y Xu Z Li J . Ferroptosis: New strategies and ideas for the treatment of pancreatic ductal adenocarcinoma. Front Biosci (Landmark Ed). (2024) 29:45. doi: 10.31083/j.fbl2901045 38287825

[11] Obeagu EI Maibouge Tanko MS . Iron metabolism in breast cancer: Mechanisms and therapeutic implications: A narrative review. Ann Med Surg (Lond). (2025) 87:3403–9. doi: 10.1097/MS9.0000000000003173 40486542 PMC12140789

[12] Tao J Yang G Zhou W Qiu J Chen G Luo W . Targeting hypoxic tumor microenvironment in pancreatic cancer. J Hematol Oncol. (2021) 14:14. doi: 10.1186/s13045-020-01030-w 33436044 PMC7805044

[13] Zhan Y Su L Lin Q Pan X Li X Zhou W . Prognostic value of serum transferrin level before radiotherapy on radio-sensitivity and survival in patients with nasopharyngeal carcinoma. J Pers Med. (2023) 13(3):511. doi: 10.3390/jpm13030511 36983693 PMC10055805

[14] Reznik E Torjani A . Mechanisms of stress-attributed breast cancer incidence and progression. Cancer Causes Control. (2024) 35:1413–32. doi: 10.1007/s10552-024-01884-2 39012513

[15] Aebi S Karlsson P Wapnir IL . Locally advanced breast cancer. Breast (Edinburgh, Scotland). (2022) 62:S58–62. doi: 10.1016/j.breast.2021.12.011 34930650 PMC9097810

[16] Chen F Fan Y Hou J Liu B Zhang B Shang Y . Integrated analysis identifies TfR1 as a prognostic biomarker which correlates with immune infiltration in breast cancer. Aging. (2021) 13:21671–99. doi: 10.18632/aging.203512 34518441 PMC8457555

[17] Li J Hao C Wang K Zhang J Chen J Liu Y . Chinese society of clinical oncology (CSCO) breast cancer guidelines 2024. Transl Breast Cancer Res. (2024) 5:18. doi: 10.21037/tbcr-24-31 39184927 PMC11341997

[18] Dasari N Guntuku GS Pindiprolu SKSS . Targeting triple negative breast cancer stem cells using nanocarriers. Discov Nano. (2024) 19:41. doi: 10.1186/s11671-024-03985-y 38453756 PMC10920615

[19] Blanco Sánchez A Yébenes L Berjón A Hardisson D . Evaluation of pathological response to neoadjuvant chemotherapy in breast cancer: Correlation with molecular phenotype. Rev Esp Patol. (2021) 54:8–16. doi: 10.1016/j.patol.2020.07.003 33455698

[20] Valenza C Trapani D Loibl S Chia SKL Burstein HJ Curigliano G . Optimizing postneoadjuvant treatment in patients with early breast cancer achieving pathologic complete response. J Clin Oncol. (2024) 42:2372–6. doi: 10.1200/JCO.23.01935 38569132

[21] Perry LM Sevilimedu V Polidorio N Abuhadra N Morrow M Plitas G . Predictors of pathologic complete response with neoadjuvant chemo-immunotherapy in early-stage triple-negative breast cancer. Ann Surg Oncol. (2025) 32:3991–4001. doi: 10.1245/s10434-025-17081-7 40025324 PMC12055475

[22] Karn T Meissner T Weber KE Solbach C Denkert C Engels K . A small hypoxia signature predicted pCR response to bevacizumab in the neoadjuvant GeparQuinto breast cancer trial. Clin Cancer Res. (2020) 26:1896–904. doi: 10.1158/1078-0432.CCR-19-1954 31932495

[23] Buschhaus JM Rajendran S Humphries BA Cutter AC Muñiz AJ Ciavattone NG . Effects of iron modulation on mesenchymal stem cell-induced drug resistance in estrogen receptor-positive breast cancer. Oncogene. (2022) 41:3705–18. doi: 10.1038/s41388-022-02385-9 35732800 PMC9288981

[24] Oany AR Upadhyay S Tsui WNT Hailemariam A Latka S Landua JD . Orphan nuclear receptor 4A1 (NR4A1) and NR4A2 are endogenous regulators of CD71 and their ligands induce ferroptosis in breast cancer. Cell Death Dis. (2025) 16:776. doi: 10.1038/s41419-025-08143-5 41184246 PMC12583510

[25] Li J He D Li S Xiao J Zhu Z . Ferroptosis: The emerging player in remodeling triple-negative breast cancer. Front Immunol. (2023) 14:1284057. doi: 10.3389/fimmu.2023.1284057 37928550 PMC10623117

[26] Yan HF Zou T Tuo QZ Xu S Li H Belaidi AA . Ferroptosis: Mechanisms and links with diseases. Signal Transduct Target Ther. (2021) 6:49. doi: 10.1038/s41392-020-00428-9 33536413 PMC7858612

